# High-fat diet-induced diabetes leads to vascular alterations, pericyte reduction, and perivascular depletion of microglia in a 6-OHDA toxin model of Parkinson disease

**DOI:** 10.1186/s12974-021-02218-8

**Published:** 2021-08-10

**Authors:** Osama F. Elabi, João Paulo M. C. M. Cunha, Abderahim Gaceb, Malin Fex, Gesine Paul

**Affiliations:** 1 Translational Neurology Group, Department of Clinical Science, Wallenberg Neuroscience Center and Wallenberg Center for Molecular Medicine, Lund University, 22184 Lund, Sweden; 2 Unit of Molecular Metabolism, Lund University Diabetes Centre, Jan Waldenströms gata 35, Box 50332, 202 13 Malmö, Sweden; 3 Department of Neurology, Scania University Hospital, 22185 Lund, Sweden

**Keywords:** Diabetes, Parkinson’s disease, Vascular alterations, Pericytes, Perivascular microglia

## Abstract

**Background:**

Diabetes has been recognized as a risk factor contributing to the incidence and progression of Parkinson’s disease (PD). Although several hypotheses suggest a number of different mechanisms underlying the aggravation of PD caused by diabetes, less attention has been paid to the fact that diabetes and PD share pathological microvascular alterations in the brain. The characteristics of the interaction of diabetes in combination with PD at the vascular interface are currently not known.

**Methods:**

We combined a high-fat diet (HFD) model of diabetes mellitus type 2 (DMT2) with the 6-OHDA lesion model of PD in male mice. We analyzed the association between insulin resistance and the achieved degree of dopaminergic nigrostriatal pathology. We further assessed the impact of the interaction of the two pathologies on motor deficits using a battery of behavioral tests and on microglial activation using immunohistochemistry. Vascular pathology was investigated histologically by analyzing vessel density and branching points, pericyte density, blood–brain barrier leakage, and the interaction between microvessels and microglia in the striatum.

**Results:**

Different degrees of PD lesion were obtained resulting in moderate and severe dopaminergic cell loss. Even though the HFD paradigm did not affect the degree of nigrostriatal lesion in the acute toxin-induced PD model used, we observed a partial aggravation of the motor performance of parkinsonian mice by the diet. Importantly, the combination of a moderate PD pathology and HFD resulted in a significant pericyte depletion, an absence of an angiogenic response, and a significant reduction in microglia/vascular interaction pointing to an aggravation of vascular pathology.

**Conclusion:**

This study provides the first evidence for an interaction of DMT2 and PD at the brain microvasculature involving changes in the interaction of microglia with microvessels. These pathological changes may contribute to the pathological mechanisms underlying the accelerated progression of PD when associated with diabetes.

**Supplementary Information:**

The online version contains supplementary material available at 10.1186/s12974-021-02218-8.

## Background

Parkinson’s disease (PD) is one of the fastest growing neurological diseases, surpassing that of Alzheimer’s disease. In 2015, PD affected 6.9 million people worldwide, a number expected to double by 2040 due to the aging population [1]. PD is pathologically characterized by the progressive degeneration of the nigrostriatal system resulting in a dopamine deficit that is clinically visible as rigidity, bradykinesia, and resting tremor [2]. All currently available treatments are purely symptomatic and limited by side effects and lack of efficacy as the disease continues to progress [3]. Perhaps the major unmet clinical need in PD of our time is the development of a therapy that can halt or slow the progression of this disease.

Although PD is associated with distinct histological changes such as the formation of Lewy bodies containing aggregated alpha-synuclein (α-syn) [4], concomitant non-cell autonomous pathological alterations of the local microenvironment are gaining importance as those might sustain or aggravate the neuronal degeneration or dysfunction. Elucidation of factors that contribute to disease progression may help to reveal the underlying mechanisms aggravating the disease and identify targets that can be addressed to slow disease progression. Numerous studies now suggest an association between neurodegeneration and metabolic diseases. Accumulating epidemiological evidence indicates that preexisting diabetes is a risk factor for developing PD [5–9] and also a negative prognostic factor for PD. In fact, diabetes mellitus type 2 (DMT2) and/or dysregulated glucose metabolism in patients with PD is associated with a more aggressive PD phenotype [5, 7, 10]. PD patients show not only a faster motor and cognitive decline in the presence of diabetes, but also reduced striatal dopamine transporter (DAT) binding [7]. Furthermore, individuals with diabetes in the absence of PD can show signs of subclinical striatal dopaminergic dysfunction on DAT scans [7], supporting a pathophysiological association between PD and diabetes. Additional evidence supporting the link between metabolic dysfunction and neurodegeneration comes from clinical and preclinical studies demonstrating a beneficial effect of anti-diabetic medication in PD [11] and PD models reviewed in [12].

A number of hypothesis as to the pathological mechanisms leading to aggravation of PD under diabetic conditions has been put forward such as defective insulin signaling, increased oxidative stress, mitochondrial dysfunction, and neuroinflammation (reviewed in [9]).

Interestingly, not much attention has been paid to the fact that DMT2 and PD both share pathological microvascular alterations in the brain. Brain vascular changes are recognized as a common denominator of several neurodegenerative disorders, such as Alzheimer’s disease [13]. In PD both, post-mortem data and cerebrospinal fluid analyses have shown changes in small blood vessels and markers of blood–brain barrier (BBB) leakage as part of the progressive brain pathology [14–17]*.* The findings in post-mortem studies are supported by preclinical animal studies in toxin-induced PD models [18, 19] and recently also in a progressive α-syn PD model [20], pointing to a temporal dynamic of vascular changes in PD with an initial compensatory angiogenesis and vascular rarefication at later stages of the disease [20].

While microvascular alterations in PD are still being fully characterized, DMT2 is well recognized for its vascular pathology. Similar to the retinal and renal complications, diabetes is associated with signs of cerebral vascular proliferation and progressive BBB disruption [21–27]. Interestingly, the basal ganglia [21] and the midbrain [22, 23] are most susceptible to diabetes-induced microvascular damage, and both regions are consistent with the localization of dopaminergic neurons/fibers affected in PD. The type of interaction between DMT2 and PD at the vascular interface, however, is not known.

Here, we investigated the effect of a 21-week exposure to a high-fat diet (HFD) in combination with a toxin-induced PD model on microvascular alterations, in particular vascular density, pericyte density, BBB leakage, and the microglial response.

## Material and methods

### Animals and experimental design

Sixty male adult C57BL/6 J mice at age of 12 weeks (purchased from Taconic Biosciences) were used in this study. Male C57BL6 mice were chosen as they develop a more severe phenotype on HFD when it comes to metabolic parameters [28]. The animals were housed in a 12-hour (h) light/dark cycle with free access to food and water. All experimental procedures were carried out in accordance with the European Directive 2010/63/EU guidelines and approved by the Ethical Committee at Lund University. Mice were fed either HFD or a control diet (CTRL) for 7 weeks before 6-hydroxydopamine (6-OHDA) toxin or vehicle infusion into the medial forebrain bundle (MFB) and maintained on the same diet throughout the study. Behavioral assessments were conducted 8–9 weeks post-lesion, and glucose tolerance test (GTT) and blood sampling were performed 10 weeks post-lesion corresponding to 17 weeks after induction of diet. The mice were sacrificed 14 weeks after the lesion, that is 21 weeks after induction of diet.

Several mice had to be excluded from the study due to either unsuccessful PD lesion, illness, or death during experimental procedures before reaching the study end point. Twenty-five mice were included in the final analysis.

### Diet specification

An HFD regime was used to induce insulin resistance mimicking DMT2 [29]. After 1 week of acclimatization, the mice were split randomly into two groups and fed with either HFD containing 60% fat (D12492, Research Diets, USA, *n* = 30) or a CTRL diet with a matched formula containing 10% fat (D12450B, Research Diets, USA, *n* = 30). Mice were kept on the respective diet for a total of 21 weeks. The effect of the diet was measured by the glucose tolerance test (GTT) and insulin levels 17 weeks after the induction of the diet.

### Glucose tolerance test (GTT) and insulin resistance index (HOMA-IR)

For the GTT, d-glucose (2 g/kg) was injected intraperitoneally (i.p.) in mice anesthetized with midazolam (0.4 mg/mouse; Dormicum®; Hoffmann-La Roche) and a combination of fluanison (0.9 mg/mouse) and fentanyl (0.02 mg/mouse; Hypnorm®; Janssen, Beerse, Belgium). Mice were fasted 4 h before the GTT and kept on a heating pad for the entire time to maintain the body temperature. Blood (30 μl) was drawn retro-orbitally at 0, 15, 30, 60, and 120 min after glucose injection. Plasma was stored at − 80 °C until analysis. Glucose and insulin levels were determined by Amplex red glucose/glucose oxidase assay kit (Invitrogen™) and insulin ELISA (Mercodia Mouse Insulin ELISA; Mercodia AB, Uppsala, Sweden), respectively. Fasting blood collected from the tolerance test at time point 0 was used to obtain an insulin resistance index (Homeostatic Model Assessment of Insulin Resistance (HOMA-IR); HOMA-IR = (Glucose_0_ in mmol/l) × (Insulin_0_ in μg/l)/22.5) [30]. The cut-off value for insulin resistance was chosen based on the 90th percentile of the HOMA-IR values of the sham/CTRL diet group [31].

### 6-OHDA lesion

Seven weeks after initiation of the diet, animals on HFD and CTRL diet were randomized to receive either 6-OHDA (lesion group) or 0.9% sodium chloride (sham group). The number of animals per group is specified in the respective figure legends. The 6-OHDA (lyophilized powder including 0.025% ascorbic acid, Sigma) was reconstituted with 0.9% saline to obtain a concentration of 1 μg/μl and stored at − 20 °C and kept on ice during the surgery. 6-OHDA or saline was infused unilaterally into the MFB using stereotaxic surgery as previously described [32]. Briefly, a Hamilton syringe was connected to a cannula (tip diameter equal 50 μm), and 1 μl of 6-OHDA solution or saline was infused into the MFB at a rate of 0.5 μl/min using the following coordinates: from Bregma: A/P =  − 1.2, M/L =  − 1.3, and D/V =  − 4.75 [33]. The cannula was left for 2 min before being slowly retracted. During surgery, mice were anesthetized with 4–5% isoflurane (IsoFlo vet, Apoteksbolaget, Sweden) in a carrier of oxygen and nitrous oxide mixture at a ratio of 2:1 for induction and then maintained at 2% isoflurane throughout the entire surgery. At the site of incision, 100 μL of Marcain (0.25 mg/mL, Apoteksbolaget, Sweden) was injected subcutaneously (s.c.) to provide local anesthesia. At the end of the surgery, the skin was sutured and mice were injected s.c. with 1 ml of saline.

### Behavioral tests

To indicate the degree of the nigrostriatal lesion, behavioral tests were performed 8 weeks after the surgical intervention corresponding to 15 weeks on the respective diet.

#### Amphetamine-induced rotation

To assess the rotational behavior, mice were placed in a glass bowl (diameter 50 cm) and harnessed to an automated rotometer to count the number and direction of the rotation as described [34]. Mice were injected with d-amphetamine (Sigma, 5 mg/kg; dissolved in sterile 0.9% saline) and allowed to acclimatize for 5 min before recording rotations. Data were obtained for 90 min and presented as the number of full-body turns per min with a positive value for ipsilateral rotation and a negative value for the contralateral rotations.

#### Corridor test

The corridor test was performed to assess the lateralized sensorimotor integration of the mice [35]. In short, the mice were first habituated for 10 min on two consecutive days before the test. The test was performed in a corridor (60 cm long, 4 cm wide, and 15 cm high) containing sucrose tablets that were scattered randomly along the floor. On the day of testing, mice were habituated for 5 min in an empty corridor and then immediately transferred to an identical corridor that contained 10 pots on each side, each containing 2–3 sucrose tables. The number of explorations made by each mouse at the ipsilateral or contralateral side was counted until a total of 20 counts was reached or 5 min had passed. The mice were put on food restriction during the habituation and the test with maintaining at 85% free-feeding body weight. The data are presented as the percentage of contralateral explorations out of the number of total explorations.

#### Cylinder test

The cylinder test was performed to assess the spontaneous forelimb lateralization. The mice were placed in a Perspex cylinder (height 20 cm, diameter 19 cm) with a mirror behind to allow a clear view. They were captured digitally by videotaping for 3 min for later analysis by a blinded examinator. The data are expressed as the percentage of weight-bearing contralateral paw touches of the cylinder wall out of the total number of touches [36].

### Tissue processing

For immunohistochemistry, mice were deeply anesthetized with sodium pentobarbital (Apoteksbolaget, Sweden) and transcardially perfused with 0.9% saline for 3 min followed by 4% paraformaldehyde (PFA) for 5 min. Brains were collected, kept in 4% PFA solution for 4 h, and then stored in a 25% sucrose solution for several days. Brains were cut coronally at 30-µm-thick sections.

### Immunohistochemistry

For immunohistochemical staining, sections were quenched with a peroxidase solution (3% H_2_O_2_, 10% methanol), diluted in phosphate-buffered saline (PBS) for 10 min before blocking for 1 h at room temperature (RT) in 3% serum diluted with 0.25% Triton X-100-PBS (PBS-TX) (Alfa Aesar). Primary antibodies diluted with 1% serum in PBS-TX were incubated overnight (O/N) at RT for rabbit anti-tyrosine hydroxylase (TH; 1:1000, Millipore) and anti-CD11b (1:200, Bio-Rad) or for 2 h at RT followed by O/N incubation at 4 °C for rat anti-dopamine transporter (DAT, 1:1000, Millipore).

Sections were then incubated for 2 h at RT with the corresponding biotinylated secondary antibodies (1:200, Vector Laboratories), followed by 1-h incubation with an avidin–biotin kit (Vectastain Elite ABC kit, Vector Laboratories), and the staining was revealed using chromogen 3,3-diaminobenzidine (DAB, Peroxidase Substrate Kit, Vector Laboratories).

For double immunofluorescence staining, sections were washed 3 times for 10 min with PBS, blocked with 5% serum in PBS-TX for 1 h at RT, and incubated with the following primary antibodies diluted with 3% serum in PBS-TX O/N at RT: goat anti-CD31 (1:200, R&D Systems) rat anti-CD13 (1:200, Bio-Rad), rabbit anti-fibrinogen (1:400, Abcam), rat anti-CD11b (1:200, Bio-Rad), or rabbit anti-mouse immunoglobulin G (IgG) (1:200, Dako).

Sections were then incubated for 1 h at RT with the respective fluorophore-tagged secondary antibodies: CY3-conjugated donkey anti-rat IgG or Alexa Fluor 647 donkey anti-rabbit diluted with 3% serum in PBS-TX (1:500; Jackson ImmunoResearch) or with an anti-goat biotinylated secondary antibody (1:200, Vector Laboratories) followed by fluorophore-conjugated streptavidin (Alexa Fluor 647, 1:500; Jackson ImmunoResearch). Sections were incubated with DAPI (4′,6-diami-dino-2-phenylindole) for 10 min, then washed and mounted on a gelatinized slide, and coverslipped with mounting medium (PVA/DABCO).

### Oil red O staining and quantification

Oil red O (ORO) staining was used to stain the lipids in the liver. A frozen liver sample was sliced to a 10-μm thickness using a cryostat (Leica CM3050E). The sections were then immediately fixed with 4% PFA solution for 10 min followed by washing with distilled water for 5 min. The slides were dipped in 60% isopropanol solution and immersed in a staining jar containing a filtered ORO dye (Sigma life science) diluted with distilled water in a ratio of 50:50 for 15 min. After that, the slides were dipped again in 60% isopropanol then passed in a double washing with distilled water for 10 min. Finally, PVA/DABCO mounting media was used to coverslip the slides. High-resolution images were obtained at 10 × magnification (Olympus BX53 microscope). The ORO^+^ lipid density was quantified using the area fraction measurement tool of ImageJ software (NIH, USA). The density was expressed as the percentage of the ORO^+^ fraction area of the image area.

### Confocal microscopy and image analysis

Confocal images were sampled from striatal sections according to AP + 0.62 to + 1.18 relative to bregma [33], using a Leica DMi8 confocal microscope. The collected images were obtained from a z-stack size of 10 µm and 1 µm step size. For maximum image projection, ImageJ software (NIH, USA) was used for z-stack image reconstruction. The same acquisition settings were applied for each image.

### Quantification of vessel density and branching points

Vessels stained with CD31 were analyzed in 2–3 striatal sections obtained from the dorsolateral striatum, whereby 2–3 images per section were acquired (image size 630 µm × 630 µm). The obtained z-stack images were reconstructed for maximum projection. Quantification of vessel density was performed using the measurement tool of the ImageJ software (NIH, USA). The density was expressed as the percentage of CD31^+^ area of the image area. Branching points of the CD31^+^ vessels were counted manually per image by counting vessel intersections on the maximum projection images that were verified to be vessel branches and not vessel overlay by reviewing the corresponding z-stacks image.

### Quantification of pericyte density

Image analysis of CD13^+^ pericytes was performed on 2–3 striatal sections whereby 2–3 images (image size 630 μm × 630 μm) per section were obtained from the dorsolateral striatum. The maximum projection image of CD13^+^ was analyzed using the area fraction measurement tool of ImageJ software (NIH, USA). The density was expressed as the percentage of CD13^+^ area of the image area. The ratio of CD13^+^ pericyte density to CD31^+^ vessel density was calculated by dividing the CD13^+^ pericyte density by the CD31^+^ vessel density.

### Extravascular fibrinogen and IgG quantification

To quantify vascular leakage, extravascular fibrinogen and immunoglobulin G (IgG) were analyzed. CD31 staining was used to delineate vessels and to identify and exclude intravascular fibrinogen or IgG. The area covered by extravascular fibrinogen or IgG was analyzed and reported as the percentage of the total image area analyzed using ImageJ (NIH, USA).

### DA neurons and fiber quantification

DA neurons labeled with TH in the substantia nigra (SN) were identified and counted at 20 × magnification at three SN sections per mouse located approximately at Bregma − 3.16 mm, − 3.40 mm, and − 3.64 mm [33, 37] using the Olympus BX53 microscope and CellSens software. The data were presented as a percentage of TH^+^ cells remaining in the ipsilateral side compared to the mean of the TH^+^ cells in the contralateral intact hemisphere of sham/CTRL diet mice corresponding to 100%.

The density of TH^+^ and DAT^+^ fibers was evaluated in 3 striatal sections per mouse located between AP + 0.62 to + 1.18 relative to bregma [33]. High-resolution images were obtained at 4 × magnification (Olympus BX53 microscope). The optical density of the striatal fibers was analyzed by ImageJ (NIH, USA) and normalized to the corpus callosum for each picture. The data is presented as percentage of fiber density in the ipsilateral side compared to the mean of the fiber density in the contralateral intact hemisphere of sham/CTRL diet mice corresponding to 100%.

### Multiplex sandwich ELISA

Leptin levels were quantified from plasma samples collected before mice were sacrificed using a mouse multiplex sandwich ELISA (Meso Scale Discovery, Metabolic hormones combo1, Gaithersburg, MD), according to the manufacturer instructions. Each sample (50 μl) was measured in triplicate, and the mean calculated. The coefficient of variation was below 20% for the assay.

### CD11b^+^ cell analysis

CD11b was used for labeling the microglial/phagocytic cells in the striatum. We analyzed the number, activation state, and localization of the microglial cells in relation to vessels.

#### CD11b^+^ cell count

Using unbiased stereology counting, the number of CD11b^+^ cells was estimated in the dorsal lateral striatum. The cells were counted in every 12th striatal section (section sampling fraction ssf = 12). A total of 3 sections of each mouse were used between AP + 0.62 to + 1.18 relative to bregma [33]. The count was performed in a Leica microscope equipped with a digital camera (Lecia MPS52) and connected to MicroBrightField stereological investigator software (Stereo Investigator MBF Bioscience V2019, US). Using a similar area for each section, the region of interest was delineated in the dorsolateral striatum using a 5 × magnification and counted at 100 × magnification. The counting frame (size 150 μm × 150 μm) was distributed randomly over grid (size 400 μm × 400 μm). The total count was calculated using optical fractionator population estimates. The accepted Gunderson coefficient of error for each animal was set at 0.08.

#### Microglial activation

Microglial activation was quantified based on the morphological changes of the cell. Activated microglia were identified by a larger cell body and shorter process and branches [38]. Modified from the previous method [39], ImageJ (NIH, USA) was used to measure cell branch density and cell body density. The ratio of branch density/cell body density was calculated as an index of microglial activation, whereby the lowest value represents the most activated microglia.

#### Microglia/vascular interaction

Distribution of microglia with respect to their perivascular location was presented in images showing microglial cells within a 20-μm distance from the vessel border by using the Distance Map plugin of ImageJ (NIH, USA). The communication between the vessels and perivascular microglia was quantified by measuring the density of interaction points between CD11b^+^ cells and CD31^+^ vessels using the colocalization plugin tool of ImageJ (NIH, USA) which was used to define and highlight the interaction points. The data is expressed as the ratio of the interaction point density to the CD31^+^ vessel density per image.

### Statistical analysis

All data were analyzed by GraphPad Prism version 8. Data were first examined for normal distribution using the Shapiro–Wilk normality test. The non-normally distributed data were transformed before the analysis: HOMA-IR, insulin, and GTT (AUC) data were transformed to log values, while fibrinogen and IgG leakage data were transformed to sqrt values. All data were analyzed by two-way ANOVA followed by a two-stage linear step-up procedure of Benjamini, Krieger, and Yekutieli test as multiple comparison test for all means [40]. For comparison of the TH^+^ cell count between the CTRL diet and HFD, a two-tailed *t*-test was used. Data were presented as mean ± standard error of the mean (SEM). Significance was considered at a *p*-value < 0.05. Correlations were performed using a simple linear regression analysis with *R*^2^ coefficient. Graphs were plotted with curves indicating a 95% confidence interval. The equation slope was considered different from zero with a *p*-value < 0.05.

## Results

### HFD does not affect the degree of the nigrostriatal lesion in a 6-OHDA PD model

**Fig. 1:**
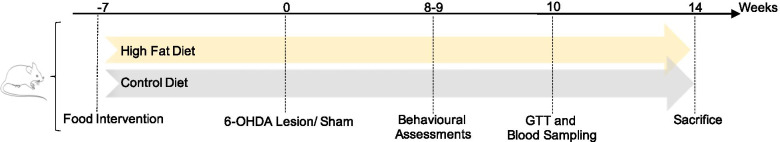
Experimental timeline. Mice were fed either a high-fat diet (HFD) or a control diet (CTRL) for 7 weeks before 6-OHDA toxin (lesion) or vehicle infusion (sham) into the medial forebrain bundle (MFB) and maintained on the same diet throughout the study. Behavioral assessments were conducted 8–9 weeks post-lesion and glucose tolerance test (GTT) and blood sampling 10 weeks post-lesion corresponding to 17 weeks after induction of diet. The mice were sacrificed 14 weeks after the lesion corresponding to 21 weeks after induction of diet

In order to induce insulin resistance and mimic DMT2, we fed mice a HFD or CTRL diet for 7 weeks before inducing a nigrostriatal lesion by administration of 6-OHDA into the MFB. Animals were then kept on the same diet for another 14 weeks before sacrifice and histological analysis (see Fig. 1 for the experimental design).

**Fig. 2:**
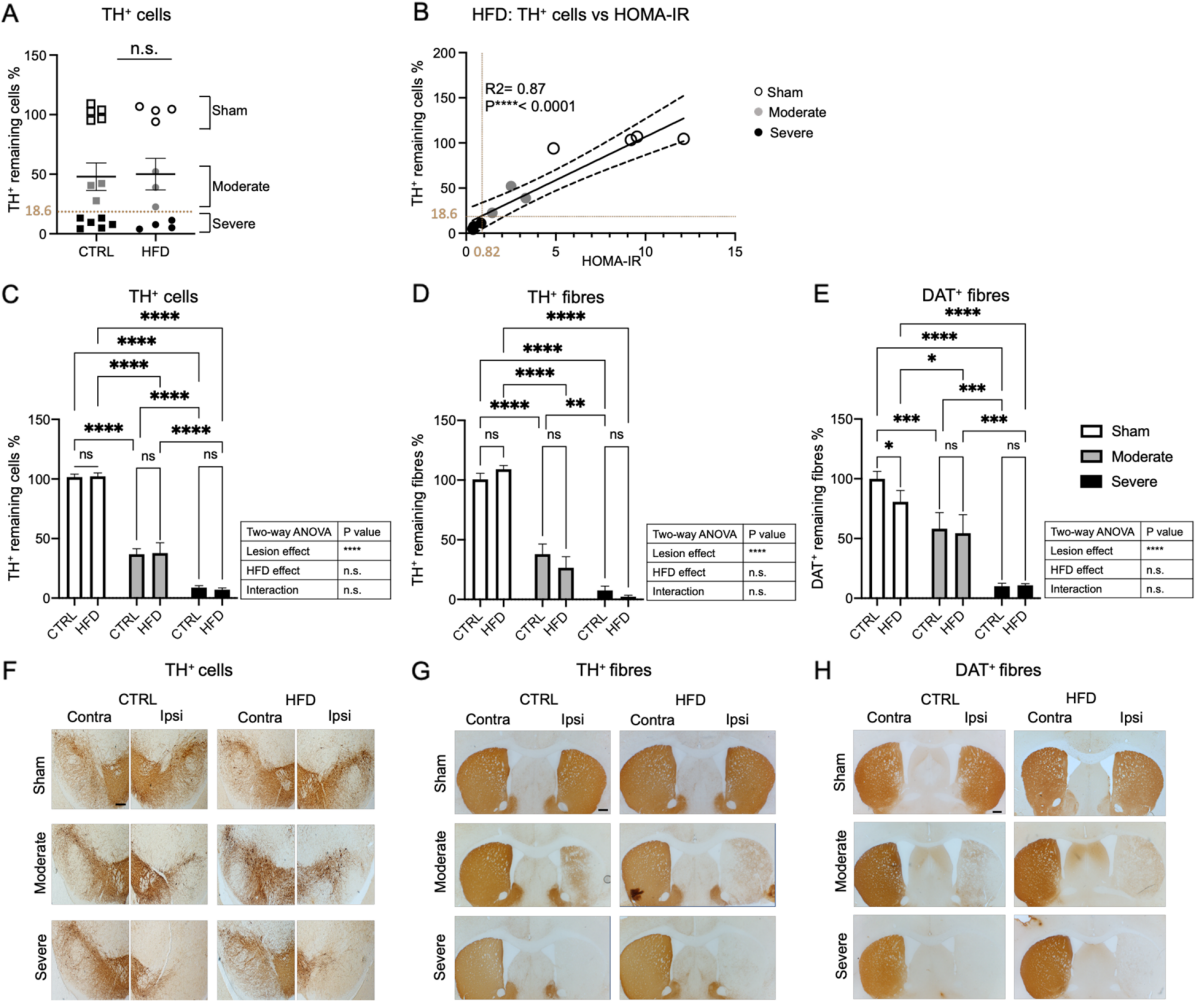
Relationship between the degree of nigrostriatal lesion and diet. **A** TH^+^ cell count in substantia nigra (SN) of sham- and 6-OHDA-lesioned mice fed either CTRL diet or HFD (CTRL diet: *n* = 14, HFD: *n* = 11). Data are expressed as percentage of remaining ipsilateral TH^+^ cells of contralateral mean TH^+^ cell count of the sham/CTRL diet group. The cut-off value between the moderate lesion and severe lesion was 18.6% of TH + remaining cells. Two-tailed Student’s *t*-test: n.s. not significant. **B** Linear regression analysis between the percentage of remaining TH^+^ cells and HOMA-IR in HFD-fed mice. The cut-off value for insulin resistance was 0.82 separating severely and moderately lesioned mice and corresponding to 18.6% remaining TH + cells. **C** TH^+^ cell count in SN depicted in the sham, moderate, and severe lesion groups fed either CTRL diet or HFD. Data are expressed as percentage of remaining ipsilateral TH^+^ cells of contralateral mean TH^+^ cell count of the sham/CTRL diet group. **D** Striatal TH^+^ and **E** striatal DAT^+^ fiber density of the sham, moderate, and severe lesion groups, fed either CTRL diet or HFD. Data are expressed as percentage of remaining ipsilateral TH^+^ or DAT^+^ fiber density of contralateral mean TH^+^ or DAT^+^ fiber density of the sham/CTRL diet group. **F** Representative brightfield images showing different degrees of dopaminergic neuron loss in SN in CTRL diet and HFD groups. **G**, **H** Representative brightfield images showing striatal TH^**+**^ and DAT^+^ fiber density in CTRL diet and HFD groups, respectively. Sham (CTRL diet: *n* = 5, HFD: *n* = 4), moderate lesion (CTRL diet: *n* = 3, HFD: *n* = 3), severe lesion (CTRL diet: *n* = 6, HFD: *n* = 4). Two-way ANOVA: **p < 0.01, ***p < 0.001, ****p < 0.0001; n.s. not significant. Scale bar 200 μm. DAT dopamine transporter, TH tyrosine hydroxylase, CTRL diet control diet, HFD high-fat diet

First, we investigated whether the type of diet had an effect on the number of TH^+^ neurons in the SN in both, sham- and 6-OHDA-lesioned mice. Even though there was some variability in both diet groups, the extent of dopaminergic neuron loss was not significantly different between HFD and CTRL diet groups (Fig. 2A).

Next, we determined whether all animals in the HFD group had developed insulin resistance and if there was a correlation between insulin resistance and the degree of nigral dopaminergic neuron loss. HOMA-IR was used as an indicator for insulin resistance, whereby the cut-off value for insulin resistance was calculated based on the 90th percentile of the HOMA-IR value of the sham/CTRL diet which was equal to 0.82 [31]. We noticed variability in the degree of insulin resistance in HFD-fed mice and therefore assessed whether there was a correlation between insulin resistance and the degree of nigral dopaminergic neuron loss. Opposite to our hypothesis, linear regression analysis showed a positive correlation between the percentage of remaining TH^+^ cells in the SN and the HOMA-IR values (*R*^2^ = 0.87, *p* < 0.001) (Fig. 2B). The animals with the most severe TH^+^ cell loss (< 18.6% remaining TH-cells) did not become insulin resistant, resulting in two subgroups of 6-OHDA-lesioned animals, those with a moderate lesion which developed insulin resistance and those with a severe lesion that did not develop insulin resistance (Fig. 2B). Consequently, we decided to analyze these two subgroups separately in this study.

Further analysis of the effect of HFD on remaining dopaminergic neurons after subgrouping the mice confirmed that only the lesion factor significantly determined the loss of the nigral dopaminergic neurons, with no main effect of the HFD or interaction between HFD and the lesion (Fig. 2C, F).

Similarly, only the lesion factor led to significantly reduced TH^+^ and DAT^+^ fiber density in the striatum, but not the diet or the interaction between the two factors (Fig. 2D, E, G, H). However, post hoc comparison showed an effect of the diet in the sham group, where HFD significantly reduced the DAT^+^ fiber density (Fig. 2E, H) consistent with previous reports showing that striatal DAT^+^ density is reduced in different diabetes models [7, 41, 42]. In both lesioned groups, this effect was not evident, possibly due to a ceiling effect in the moderate lesion group as the effect is dominated by the lesion factor and/or the absence of insulin resistance in the severe lesion group (Fig. 2D, E, G, H).

### HFD resulted in obesity and insulin resistance based on the degree of nigral cell loss

**Fig. 3:**
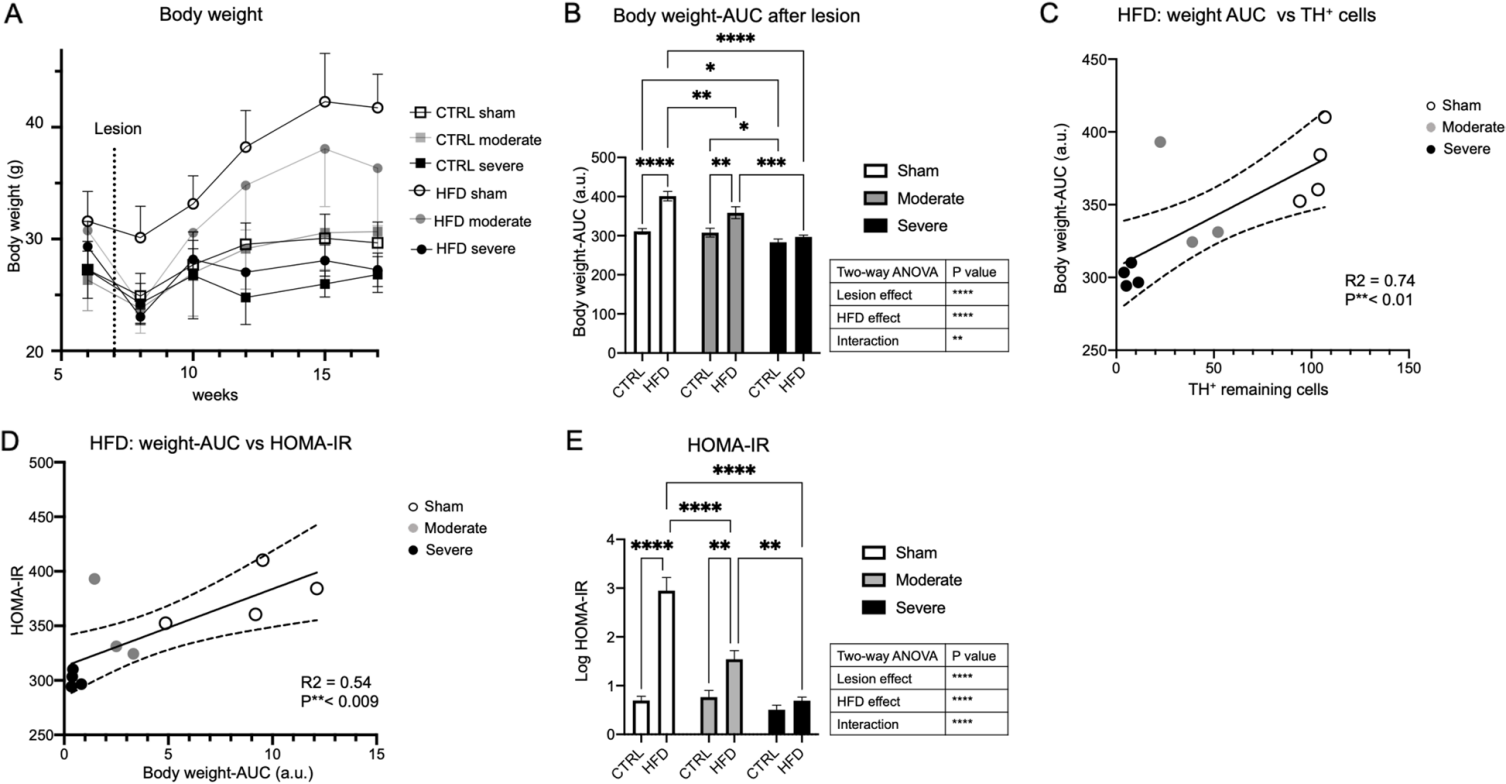
Body weight gain in relation to the degree of nigrostriatal lesion and HOMA-IR. **A** Total body weight prior to and following the 6-OHDA toxin or vehicle administration in sham, moderate, and severe lesion groups fed with either CTRL diet or HFD. **B** Area under the curve (AUC) of body weight for 11 weeks post-lesion of mice fed with either CTRL diet or HFD in sham, moderate, and severe lesion groups. Data are expressed in (a.u). **C** Linear regression analysis between AUC of body weight and remaining TH^+^ cells or between **D** the HOMA-IR value and AUC of body weight in HFD group. **E** Histogram showing log HOMA-IR value in the sham, moderate, and severe lesion groups for CTRL diet and HFD. Sham (CTRL diet: *n* = 5, HFD: *n* = 4), moderate lesion (CTRL diet: *n* = 3, HFD: *n* = 3), severe lesion (CTRL diet: *n* = 6, HFD: *n* = 4). Two-way ANOVA (**B**, **E**): *p < 0.05, **p < 0.01, ***p < 0.001, ****p < 0.0001; linear regression analysis (**C**, **D**): **p < 0.01. HOMA-IR Homeostatic Model Assessment of Insulin Resistance, AUC area under the curve, a.u arbitrary unit

The ability of HFD to induce insulin resistance is usually associated with weight gain [24, 26, 43]. We next investigated whether Parkinsonian mice might have failed to gain weight and therefore did not develop insulin resistance. Mice from all experimental groups lost weight after the surgery. As expected, the sham/HFD group gained weight over time, whereas weight gain in the moderate lesion group was less, and mice in the severe lesion group failed to gain weight over time (Fig. 3A). This was further reflected in the analysis of the area under the curve (AUC) of the body weight gain after surgery. The HFD, lesion, and the interaction between both had a significant effect on the body weight. As expected, HFD induced obesity in the sham mice compared to the CTRL diet. HFD also increased body weight compared to CTRL diet in moderate lesion mice, but there was no significant difference in weight between HFD and CTRL diet in severe lesion mice (Fig. 3B). Notably, the increase in body weight in the sham group was significantly higher compared to the moderate lesion group (*p* = 0.01) (Fig. 3B).

Indeed, weight gain positively correlated with the percentage of remaining nigral TH^+^ cells (*R*^2^ = 0.74, *p* < 0.001) (Fig. 3C), likely due to a lower food intake in the severe lesion group, and weight gain correlated with the degree of insulin resistance (*R*^2^ = 0.54, *p* < 0.009) (Fig. 3D). The HFD led to significantly higher HOMA-IR values in the sham and the moderate lesion group, but not in the severe lesion group (Fig. 3E). Similarly, HFD significantly increased the GTT-AUC and fasting glucose levels in sham and moderate lesion groups, but not in severe lesion mice (Additional Figure 1A, B).

The level of fasting insulin was only significantly increased in the sham/HFD group (Additional Figure 1C). Further analysis for plasma leptin level and fat accumulation in the liver showed that HFD increased fat accumulation and leptin levels in the sham group but not in the severe lesion/HFD group. There was a significant increase in leptin and a trend toward increased liver fat accumulation in the moderate lesion group, even though there were only low numbers of liver and plasma samples available to analyze in this group (Additional Figure 2).

In summary, these analyses confirmed that HFD induced different degrees of insulin resistance and body weight gain in the experimental groups with higher insulin resistance and obesity in the sham group and moderate insulin resistance and obesity in moderate lesion mice, while no insulin resistance or obesity was achieved in the severe lesion group.

However, the moderate lesion group with remaining dopamine neurons of about 36.8% developed an impaired glucose tolerance with mild insulin resistance and is likely most representative of the clinical scenario of PD patients who have impaired glucose tolerance or DMT2.

### HFD partially further aggravated the motor impairment of moderately and severely lesioned mice

**Fig. 4:**
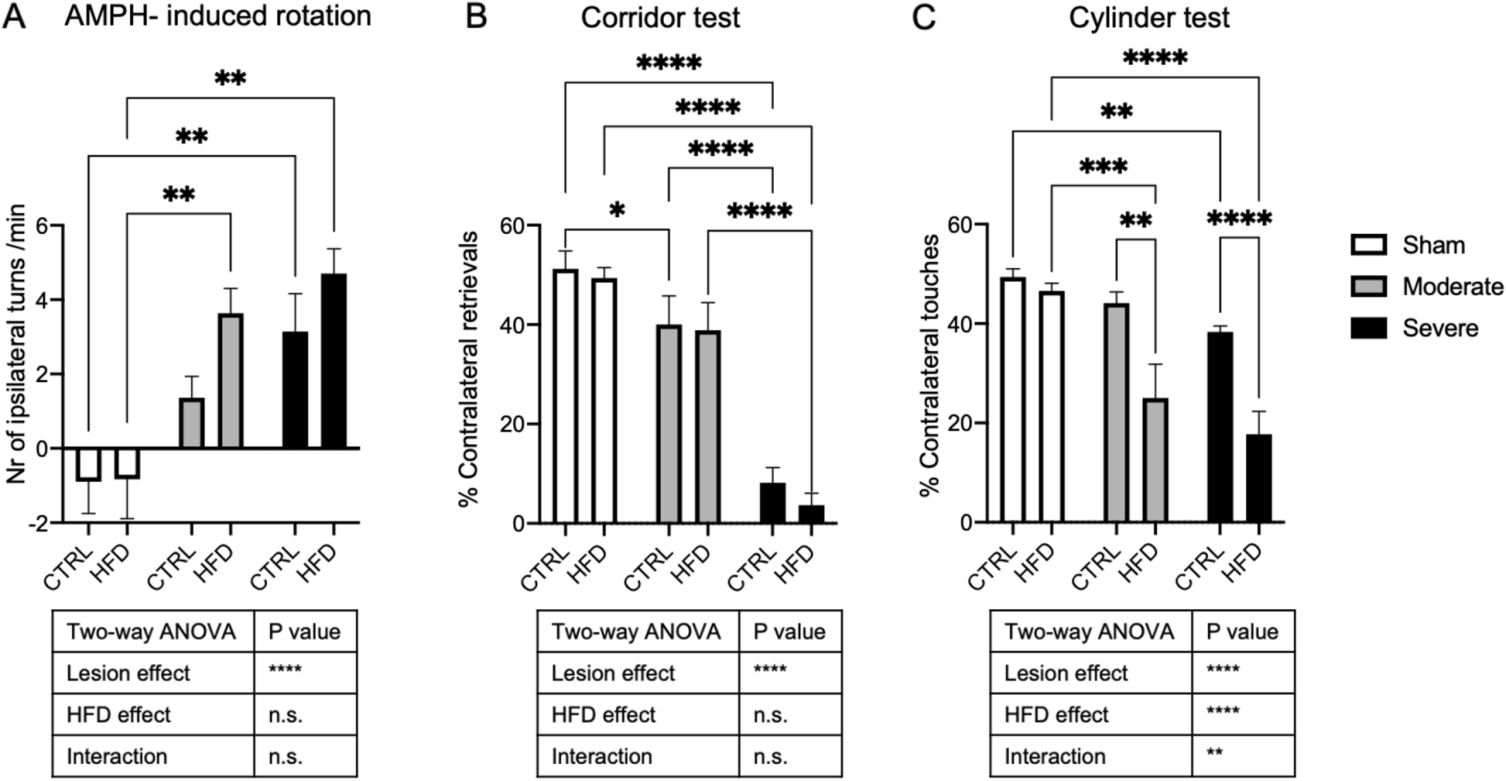
Effect of HFD on motor impairment. **A** Amphetamine-induced rotation test, **B** corridor test, and **C** cylinder test of sham, moderate, and severe lesion groups on CTRL diet and HFD. Data are expressed in number of ipsilateral turns/min, percentage of contralateral retrievals, and percentage of contralateral touches, respectively. Sham (CTRL diet: *n* = 5, HFD: *n* = 4), moderate lesion (CTRL diet: *n* = 3, HFD: *n* = 3), severe lesion (CTRL diet: *n* = 6, HFD: *n* = 4). Two-way ANOVA: *p < 0.05, **p < 0.01, ***p < 0.001, ****p < 0.0001; n.s. not significant

To assess the impact of HFD on motor activity, we evaluated the behavior of the different groups using amphetamine-induced rotation, corridor, and cylinder tests. HFD had no impact on motor performance in the sham animals in any of the motor tests. As expected, the 6-OHDA lesion significantly increased amphetamine-induced rotations (*p* < 0.0001) and reduced contralateral retrievals in the corridor test (*p* < 0.0001). There was a clear trend toward worse performance in amphetamine-induced rotations of both lesioned groups when on HFD compared to when on CTRL diet; however, this did not reach significance (*p* = 0.14, *p* = 0.21, respectively) (Fig. 4A, B). In contrast, the lateralization in the cylinder test was significantly increased by HFD, lesion, and the interaction (lesion, *p* < 0.0001; HFD, *p* < 0.0001; interaction, *p* = 0.006) (Fig. 4C). In both, moderate and severe lesion mice, HFD significantly reduced the number of contralateral touches compared to the CTRL diet (*p* = 0.0011; *p* < 0.001, respectively) (Fig. 4C). Our findings indicate that HFD impacts on motor performance in the PD model in the cylinder test.

### HFD and 6-OHDA lesion induced vascular and pericyte alterations at the striatum

Microvascular alterations are a common denominator of several neurodegenerative disorders [44] and have been described as part of the pathology in PD [18, 20, 45]. Similarly, DMT2 leads to vascular alterations also in the brain [25], and the effect of DMT2 on the brain microvasculature in PD models, however, is not known.

We therefore next analyzed the vessel density and branching (CD31) as well as the pericyte density (CD13) in the ipsilateral striatum of mice in all experimental groups using immunohistochemistry.

**Fig. 5:**
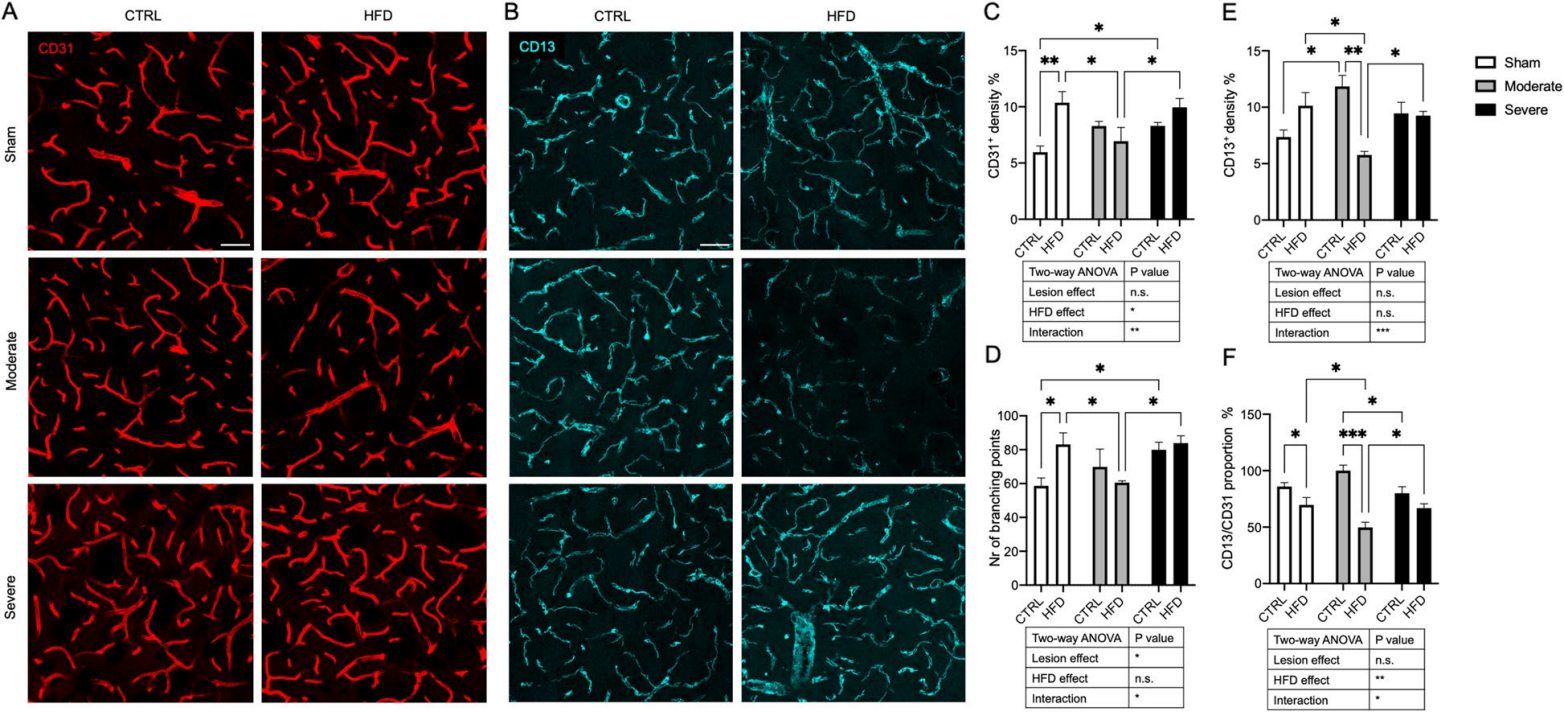
HFD effect on striatal vessel density, branching points, and pericyte density. **A** Confocal images showing CD31^+^ vessel density (red) and **B** CD13^+^ pericyte density (cyan) in sham, moderate, and severe lesion groups fed with either CTRL diet or HFD. **C** Quantification of vessel density expressed in percentage of the total image area and **D** vessel branching points in all groups. **E** Quantification of CD31 density expressed in percentage of the total image area. **F** Histogram showing proportion of CD13^+^ pericyte density to CD31^+^ vessel density in percentage. Sham (CTRL diet: *n* = 5, HFD: *n* = 4), moderate lesion (CTRL diet: *n* = 3, HFD: *n* = 3), and severe lesion (CTRL diet: *n* = 6, HFD: *n* = 4). Two-way ANOVA: *p < 0.05, **p < 0.01, ***p < 0.001. Scale bar 50 μm

In sham mice, HFD significantly increased the vessel density and branching compared to the CTRL diet, indicating an angiogenic effect (Fig. 5A, C, D). However, this angiogenesis was not followed by a corresponding increase in pericyte density. This resulted in a significant reduction of the proportion of pericyte density to vessel density in HFD compared to the CTRL diet, indicating an angiogenesis with pericyte mismatch (Fig. 5B, E, F).

In moderate lesion animals on CTRL diet, there was a clear trend toward an increase in vessel density and number of branching points that became significant in severe lesion mice. The lesion also induced an increase in pericyte density that reached significance in moderate lesion mice compared to sham/CTRL diet mice, indicating an angiogenic response to the lesion but no pericyte mismatch in response to a 6-OHDA lesion (Fig. 5E, F).

We next analyzed the effect of HFD in combination with the 6-OHDA lesion. In severe lesion mice, HFD did not add a significant angiogenic effect to the lesion effect as assessed by vessel density and number of branching points and did not significantly change the pericyte density or vessel/pericyte ratio, likely due to the lack of insulin resistance in these animals.

In contrast to sham/HFD mice, in moderate lesion mice, HFD did not further increase the angiogenic effect as there was no change in the vessel density or branching compared to moderate/CTRL diet mice. Vessel density and number of branching points were significantly less in moderate/HFD mice compared to sham/HFD mice. Strikingly, HFD in moderate lesion mice leads to a significant reduction in pericyte density in these animals and a reduced vessel/pericyte ratio compared to when mice were fed a CTRL diet, suggesting a possible pericyte dysfunction in the moderate/HFD group (Fig. 5A–F).

In summary, even though HFD has an angiogenic effect with pericyte mismatch in sham-lesioned animals, the combined effect of HFD and moderate 6-OHDA lesion did not stimulate angiogenesis but resulted in a significant reduction of pericytes in the insulin-resistant mice compared to sham/HFD mice and moderate/CTRL diet mice.

### HFD and PD lesion impact on number, activation and vessel interaction of microglia in the striatum

To investigate other potential factors that may contribute to the pathological interaction between DMT2 and PD, we investigated the microglial response in the ipsilateral striatum using CD11b staining.

**Fig. 6:**
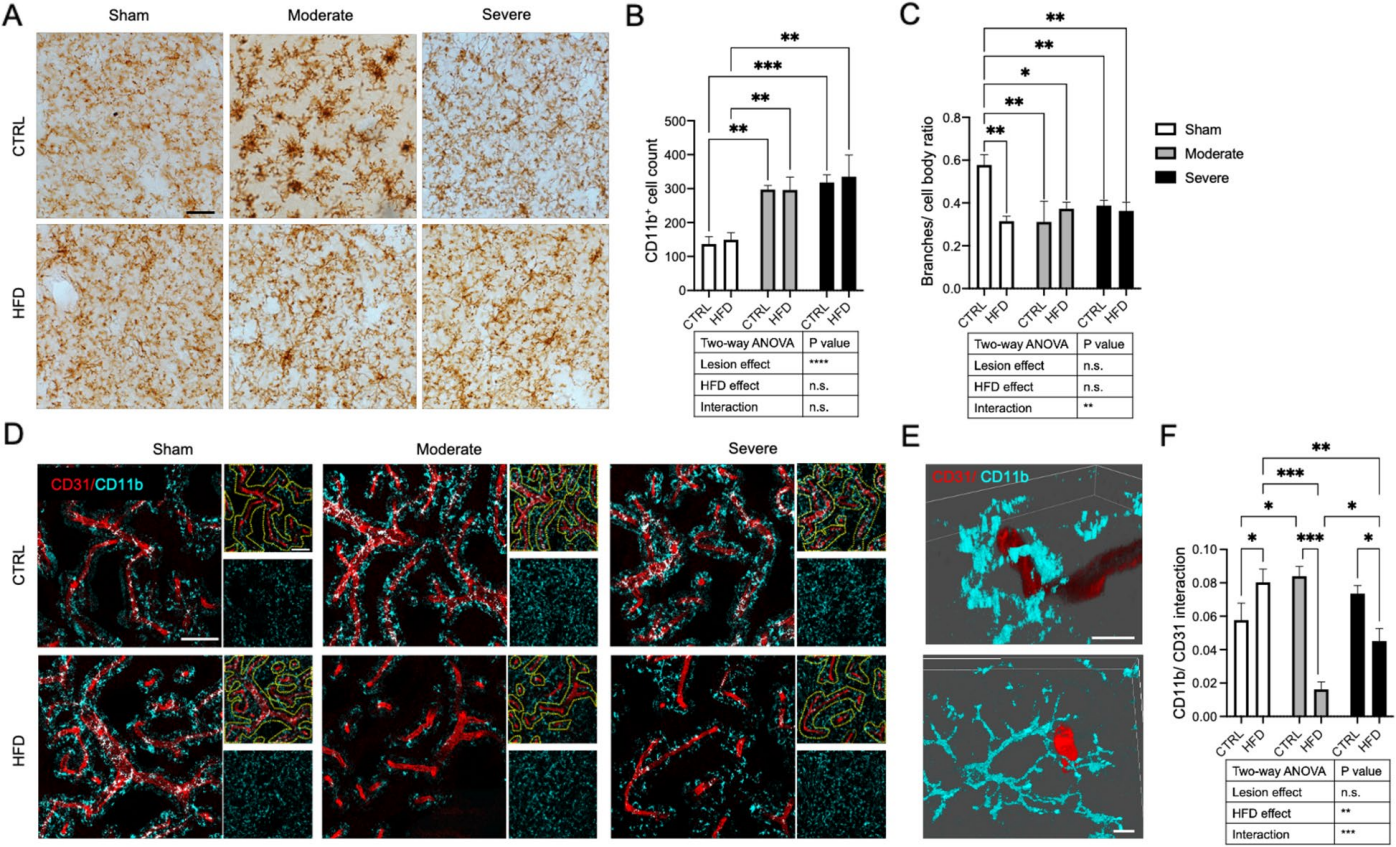
HFD effect on striatal CD11b^+^ cells of the sham, moderate, and severe lesion mice. **A** Brightfield images showing morphology and numbers of CD11b^+^ cells in sham, moderate, and severe lesion groups fed with either CTRL diet or HFD. **B** Quantification of striatal CD11b^+^ cell numbers in sham, moderate, and severe lesion groups fed with either CTRL diet or HFD. **C** Activation of CD11b^+^ cells presented as a ratio of branch density/cell body density in sham, moderate, and severe lesion groups fed with either CTRL diet or HFD. A lower ratio indicates higher activation. **D** Large confocal images showing the only location of CD11b^+^ cells (cyan) around the CD31^+^ vessels (red) within 20 μm distance from the vessel. Small confocal images showing the distribution of all CD11b^+^ cells around CD31^+^ vessels delineated with a dotted line indicating 20 μm distance from the vessel (upper image) or only CD11b^+^ cells (lower image). **E** 3D confocal representative images taken from the sham/HFD group illustrating the association between CD11b^+^ cells and CD31^+^ vessels, the upper image shows CD11b^+^ cell enwrapping CD31^+^ vessel; the lower image shows CD11b^+^ cell extending branches toward the CD31^+^ vessel. **F** Histogram showing the ratio of CD11b^+^ cell interaction points with CD31^+^ vessel density of total vessel density. Sham (CTRL diet: *n* = 5, HFD: *n* = 4), moderate lesion (CTRL diet: *n* = 3, HFD: *n* = 3), and severe lesion (CTRL diet: *n* = 6, HFD: *n* = 4). Two-way ANOVA: * *p* < 0.05, **p < 0.01, ***p < 0.001. Scale bar: brightfield images, 20 μm; confocal images (large and small) 2D, 50 μm; 3D, 5 μm

We found that the number of CD11b^+^ cells increased by the effect of the lesion only, with no effect of the HFD or interaction between the HFD and the lesion (Fig. 6A, B). We next quantified microglial activation by dividing the microglial branch density by the cell body size. Morphologically, the activated microglia have a larger cell body and fewer branches [38], resulting in a lower value of the ratio that indicated the more activated CD11b^+^ cell. There was a microglial activation in the sham/HFD group and both lesion groups fed with either CTRL diet or HFD compared to the sham/CTRL diet group (Fig. 6C). However, we noticed differences in the distribution of activated microglia especially with respect to their perivascular location (Fig. 6D). Therefore, we further investigated the interaction between the CD11b^+^ cells and blood vessels by quantifying the interaction points between the CD11b^+^ cells and the CD31^+^ vessels.

In sham mice, the analysis showed that the HFD significantly increased the microglial/vessel interaction compared to the CTRL diet (Fig. 6D, F). Also, the 6-OHDA lesion/CTRL diet increased the interaction between microglia and vessels, but this reached only significance in the moderate lesion group (Fig. 6D, F).

Interestingly, in contrast to its effect on sham mice, HFD in combination with a 6-OHDA lesion led to significantly reduced interaction between microglia and blood vessels in both, moderate and severe lesion mice. The interaction was significantly less in the moderate/HFD compared to the severe/HFD group, likely due to the differences in insulin resistance between these two groups (Fig. 6D, F). Further analysis showed that the CD11b/CD31 interaction positively correlated with CD13^+^ pericyte density (Additional Figure 3), indicating a possible role of pericytes in attracting the microglia toward the vessels.

### HFD affects the BBB integrity in the striatum of sham and the severe lesion mice

We next investigated whether the observed vascular and microglial changes were reflected in BBB leakage by assessing the extravasation of large plasma proteins like fibrinogen and IgG in the ipsilateral striatum.

**Fig. 7:**
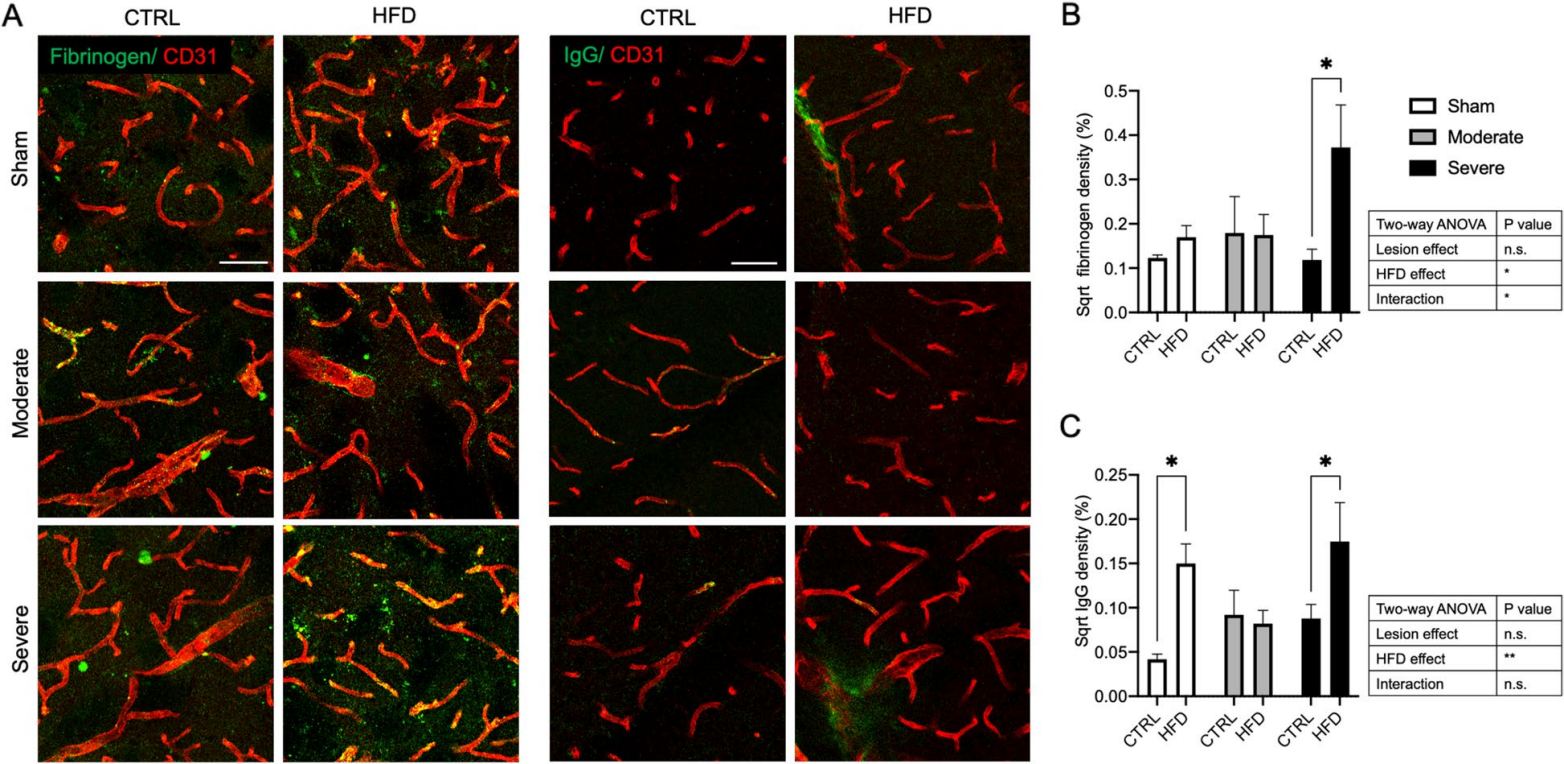
HFD effect on BBB integrity in the striatum of sham, moderate, and severe lesion groups. **A** Confocal images showing plasma protein leakage (green) (fibrinogen left panel and IgG right panel) in sham, moderate, and severe lesion groups fed with either CTRL diet or HFD. **B**, **C** Corresponding quantification of extravascular fibrinogen and IgG, respectively. Data are expressed in sqrt % of leakage fraction area of the total image area. Sham (CTRL diet: *n* = 5, HFD: *n* = 4), moderate lesion mice (CTRL diet: *n* = 3, HFD: *n* = 3), and severe lesion mice (CTRL diet: *n* = 6, HFD: *n* = 4). Two-way ANOVA: *p < 0.05, **p < 0.01. Scale bar 50 μm. IgG immunoglobulin G

The analysis of fibrinogen leakage indicated that there was a main effect of the HFD and also the interaction between HFD and lesion to induce vascular leakage of fibrinogen. The post hoc pair-wise comparison showed that HFD induced the leakage only at the severe lesion group compared to the CTRL diet (*p* = 0.012) (Fig. 7A, B). Analysis of IgG leakage revealed an effect for HFD only. The pair-wise comparison demonstrated here that HFD induced IgG leakage only in the sham and severe lesion groups, but there was no effect in the moderate lesion group when compared to the CTRL diet (Fig. 7A, C).

## Discussion

Here, we describe for the first time a number of brain microvascular alterations observed as an effect of HFD in PD, combining a mouse model of DMT2 with a MFB 6-OHDA lesion model of PD. Even though HFD did not increase nigrostriatal pathology in this PD model, it partially aggravated the motor deficit in lesioned mice. Importantly, in contrast to DMT2 or PD pathology separately resulting in different degrees of angiogenesis, this is the first report showing that a moderate PD pathology combined with HFD results in significant pericyte loss and a decrease in microglial/vascular interaction, likely accelerating the timeline of pathological microvascular changes in PD.

Here, we used a 21-week HFD in order to induce glucose intolerance and insulin resistance as a model of DMT2 [29]. As expected, the HFD induced insulin resistance and obesity in non-parkinsonian mice confirming previous studies [24–26]. However, the degree of insulin resistance achieved in moderately 6-OHDA-lesioned mice was lower and could not be achieved when mice were severely lesioned, consistent with lower or absent weight gain in these animals after the lesion. Our data indicate that modeling DMT2 in PD models is strongly influenced by variations in weight gain due to the nigrostriatal lesion. Monitoring food intake would have added additional verification of the interference of the degree of PD pathology with weight gain and consequently insulin resistance. However, the moderate PD lesion resulted in impaired glucose tolerance and mild insulin resistance, consistent with similar studies in rats [46]. This moderate loss of dopaminergic neurons and fibers reflects the clinical situation of PD patients [47]. DMT2 is now recognized as a risk factor for PD [5–7] suggesting that moderate lesion PD models with impaired glucose tolerance or DMT2 are most clinically relevant for early PD with DMT2 [48].

In our study, we did not find an aggravation of the dopaminergic cell or fiber loss in the PD model in combination with HFD. This is in contrast to previous reports where different PD models or diabetic models were used [42] and likely explained by the fact that the 6-OHDA PD model we utilized is an acute rather than slowly progressive model [49] making it less likely to observe the impact of HFD on slowly progressive nigrostriatal changes. Both the acute PD model we used and the lack of sufficient weight gain after the lesion and the resulting variation in insulin resistance may explain that we did not detect significant changes in nigrostriatal pathology in animals on HFD. However, HFD significantly aggravated the motor performance in the cylinder test and showed a trend toward higher rotations in amphetamine-induced rotations, indicating an imbalance of dopamine release between the lesioned and intact side of the brain [34]. Notably, also, animals in the HFD/severe lesion group performed worse in the cylinder test even though they were not insulin resistant 10 weeks post-lesion. It is conceivable that the exposure to HFD for 7 weeks prior to the 6-OHDA lesion was sufficient to induce microenvironmental changes in the striatum that affected the dopamine release. HFD has previously been reported to reduce striatal dopamine content [46, 50, 51], and our findings reflect the clinical data reporting that PD patients with DMT2 have higher motor scores [5, 7, 48].

In our study, we particularly investigated microvascular changes when combining HFD with PD pathology. Caused by DMT2 alone, we observed indicators of angiogenesis together with a lack of pericyte increase resulting in a mismatch between vessel and pericyte density and confirming the current knowledge of pathological angiogenesis in DMT2 [52, 53]. Pathological angiogenesis with reduced pericyte coverage is well known to be associated with vessel leakage [54, 55], confirmed by our findings of IgG leakage as an indicator of BBB leakage. We did not detect fibrinogen leakage in this group, probably due to the fact that fibrinogen has a larger molecular weight compared to IgG [24, 25].

Vascular dysfunction is now recognized as a common denominator of neurodegenerative disorders, including PD. The 6-OHDA lesion in our study resulted in a clear trend toward an increase in vessel density and the number of branching points that became significant in more severely lesioned animals. However, in contrast to the sham/HFD condition, the pericyte density was also increased. Microvascular changes have previously been shown also in 6-OHDA PD models [18, 45, 56], and we have recently reported pericyte changes, vascular alterations, and BBB leakage in an α-syn model of PD [20]. Unlike other studies, we did not detect extravasation of fibrinogen or IgG in the lesion/CTRL diet groups, possibly due to the fairly large size of the proteins studied in our experimental set up. In addition, even though there were differences in pericyte density between the moderate and severe lesion animals, in both lesion groups, there was no pericyte mismatch which might account for the absence of vascular leakage in this model.

In contrast to each condition separately, the combination of HFD and moderate PD led to significant pericyte depletion and a reduced pericyte/vessel ratio indicating pericyte dysfunction. This was not observed in severely lesioned animals, likely due to the fact that these animals were not insulin resistant.

The pericyte loss in moderate/HFD PD may counteract the angiogenic effect of DM. In an α-syn PD model, we have recently reported dynamic vessel changes from first an angiogenic response, that is likely compensatory, followed by vascular regression at more severe stages as a sign of failure of the angiogenic attempt. The vascular alterations were accompanied by a pathological alteration of pericytes already at an early stage of the disease [20].

Besides microvascular changes, inflammation is a common feature of both, DMT2 and PD [27, 57]. The increase in the number of microglial cells was only driven by the PD lesion, and not by the diet or interaction of these two factors. However, even though HFD alone did not result in an increase in the number of microglial cells, the microglial cells were significantly activated in all groups compared to sham/CTRL.

When we further characterized the perivascular microglia, we noticed a significant effect of the interaction of diet and lesion. Activated microglial cells were more located around blood vessels as a result of both DMT2 and a PD lesion leading to an increase in the connections between the vessels and microglial cells. This was evident by either increased branching or complete enwrapping of microglial cells of the vessel suggesting an attraction of microglial cells to the vessels. Similar to our findings, Haruwaka et al. showed an increase in microglial location around the vessels without change in total microglial numbers upon systemic inflammatory stressors [58]. Strikingly, in the moderate lesion/HFD group, we observed not only a clear decrease in pericyte density, but also a dramatic reduction in the interaction between the vessels and the microglia despite the fact that the number of microglial cells was increased by the lesion.

The reason for the differences in microglial/vascular interaction is not entirely clear. Microglial-vascular interaction and pericyte density were highly correlated in our study. Pericytes are well known to release several immune mediators including cytokines and chemokines in addition to phagocytic/endocytic receptors that can attract and recruit inflammatory cells [59, 60]. It is conceivable that the reduction and/or dysfunction of pericytes contributes to the reduced attraction of microglia to the blood vessels. This perivascular microglial interaction may contribute to the angiogenic process. Activated microglia causes the release of vascular endothelial growth factor-A (VEGF-A) and platelet-derived growth factor-BB (PDGF-BB) which both play an important role to enhance angiogenesis and endothelial cell proliferation [61]. It has been suggested that activated microglial cells induce vascular leakage via the release of inflammatory cytokines [62] or by phagocytosis of vessels [58, 63]. A recent study demonstrated that vascular leakage due to an inflammatory pathological stressor is dependent on perivascular activated microglia and correlates with their time of contact with the blood vessels [58]. Although there was a mismatch between the vessel and pericyte density in moderate/HFD mice, there was a significantly reduced microglia/vessel interaction possibly underlying the absence of BBB leakage in this group.

It is conceivable that the presence of HFD inhibits the attempt of compensatory angiogenesis seen in PD and accelerates the vessel changes toward a later stage of vascular regression. We have recently described those dynamic vessel changes in PD ranging from a likely compensatory angiogenic response to vascular regression at more severe stages as a sign of failure of the angiogenic attempt [14, 16, 64, 65]. Even though the diet had no impact on insulin resistance, weight gain, or liver changes in the severe group, we observed a significant effect on the interaction of microglia with the vasculature and an increased permeability of the BBB in this group. It is conceivable that an exposure for 7 weeks of HFD prior to the 6-OHDA lesion was sufficient to trigger this pathology in combination with a severe PD lesion despite that the diabetic state was not sustained due to weight loss when analyzed 10 weeks after the lesion.

## Conclusion

Here, we described the first evidence for an interaction between HFD and PD at the microvascular interface. Our finding supports that diabetes induces vascular alterations in PD that may participate in the aggravation of the motor deficit of PD. We demonstrated that HFD in moderate 6-OHDA lesion mice resulted in a significant depletion of pericytes, and reduced interactions between microvessels and perivascular microglia associated with a lack of the angiogenic response that is usually seen in PD models without HFD. Further studies are warranted to confirm these findings in also female mice and in different animal models and investigate the detailed mechanism underlying these changes.

## Supplementary Information

**Additional file 1: Figure 1.** GTT, glucose and insulin plasma level in all groups. (A) AUC of the GTT of sham, moderate and severe lesion group fed with either CTRL diet or HFD. Data are expressed as log GTT (AUC). (B, C) Fasting plasma glucose and insulin levels, respectively, of sham, moderate and severe lesion group fed with either CTRL diet or HFD. Glucose data are expressed as mmol/ L; insulin data are expressed as log insulin (µg/L). Sham (CTRL diet: *n*=5, HFD: *n*=4), moderate lesion (CTRL diet: *n*=3, HFD: *n*=3) and severe lesion (CTRL diet: *n*=6, HFD: *n*=4). Two-way ANOVA: p* <0.05, p**<0.01, p***<0.001, p****<0.0001. GTT = glucose tolerance test, AUC = area under the curve.

**Additional file 2: Figure 2.** Diet effect on the lipid content in the liver and plasma leptin level. (A) Bright field images showing ORO+ lipid staining in the liver of sham, moderate and severe lesion group fed with either CTRL diet or HFD. (B) corresponding quantification of ORO density in the liver. Data are expressed in % of total image area. Sham (CTRL diet: *n*=4, HFD: *n*=3), moderate lesion (CTRL diet: *n*=1, HFD: *n*=1) and severe lesion (CTRL diet: *n*=5, HFD: *n*=3). (C) Plasma leptin level of sham, moderate and severe lesion group fed with either CTRL diet or HFD. Data are expressed as pg/ml. Sham (CTRL diet: *n*=3, HFD: *n*=4), moderate lesion (CTRL diet: *n*=1, HFD: *n*=3) and severe lesion (CTRL diet: *n*=3, HFD: *n*=3). Two-way ANOVA: p* <0.05, p**<0.01, p***<0.001, p****<0.0001.

**Additional file 3: Figure 3.** Linear regression analysis between the CD11b+/CD31+ interaction density and the CD13+ pericyte density of sham, moderate and severe lesion group fed with either CTRL diet or HFD. Sham mice (CTRL diet: *n*=5, HFD: *n*=4), moderate lesion (CTRL diet: *n*=3, HFD: *n*=3) and severe lesion (CTRL diet: *n*=6, HFD: *n*=4). p**<0.01

## Data Availability

The datasets used and/or analyzed during the current study are available from the corresponding author on reasonable request.

## Abbreviations

6-OHDA: 6-Hydroxydopamine
AUC: Area under the curve
BBB: Blood–brain barrier
CTRL diet: Control diet
DAB: 3,3-Diaminobenzidine
DAT: Dopamine transporter
DMT2: Diabetes mellitus type 2
GTT: Glucose tolerance test
HFD: High-fat diet
HOMA-IR: Homeostatic Model Assessment of Insulin Resistance
IgG: Immunoglobulin G
MFB: Medial forebrain bundle
ORO: Oil red O
PBS: Phosphate-buffered saline
PD: Parkinson’s disease
PDGF-BB: Platelet-derived growth factor-BB
PFA: Paraformaldehyde
TH: Tyrosine hydroxylase
VEGF-A: Vascular endothelial growth factor-A
