# Supplementary material for: High-fat diet-induced diabetes leads to vascular alterations, pericyte reduction, and perivascular depletion of microglia in a 6-OHDA toxin model of Parkinson disease
Source: J Neuroinflammation. 2021 Aug 10;18:175. doi: 10.1186/s12974-021-02218-8 (PMC8353816; doi:10.1186/s12974-021-02218-8)
Supplement: Supplementary file 1 — Additional file 1: Figure 1. GTT, glucose and insulin plasma level in all groups. (A) AUC of the GTT of sham, moderate and severe lesion group fed with either CTRL diet or HFD. Data are expressed as log GTT (AUC). (B, C) Fasting plasma glucose and insulin levels, respectively, of sham, moderate and severe lesion group fed with either CTRL diet or HFD. Glucose data are expressed as mmol/ L; insulin data are expressed as log insulin (µg/L). Sham (CTRL diet: n=5, HFD: n=4), moderate lesion (CTRL diet: n=3, HFD: n=3) and severe lesion (CTRL diet: n=6, HFD: n=4). Two-way ANOVA: p* <0.05, p**<0.01, p***<0.001, p****<0.0001. GTT = glucose tolerance test, AUC = area under the curve. [file 12974_2021_2218_MOESM1_ESM.pdf]

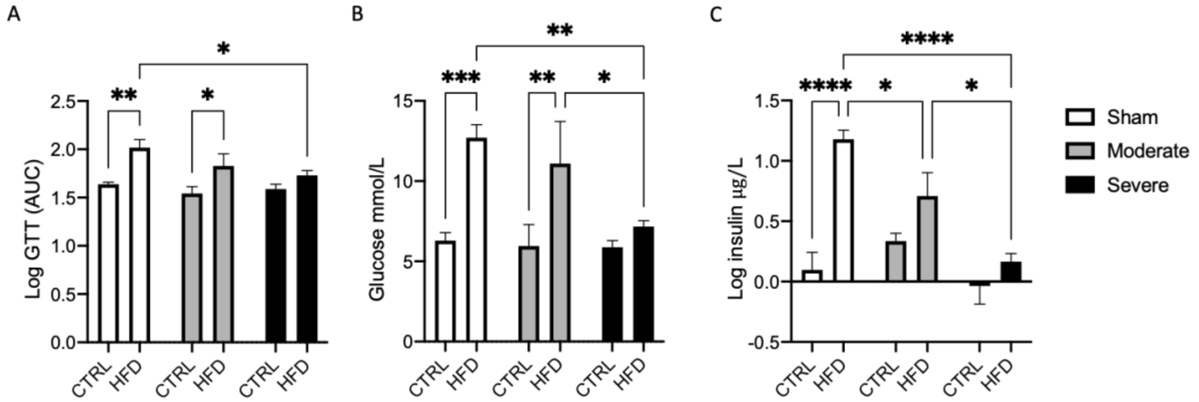

Two-way ANOVA	P value
Lesion effect	*
HFD effect	***
Interaction	n.s.

Two-way ANOVA	P value
Lesion effect	*
HFD effect	****
Interaction	*

Two-way ANOVA	P value
Lesion effect	***
HFD effect	****
Interaction	**

Additional figure 1: GTT, glucose and insulin plasma level in all groups. (A) AUC of the GTT of sham, moderate and severe lesion group fed with either CTRL diet or HFD. Data are expressed as log GTT (AUC). (B, C) Fasting plasma glucose and insulin levels, respectively, of sham, moderate and severe lesion group fed with either CTRL diet or HFD. Glucose data are expressed as mmol/ L; insulin data are expressed as log insulin (µg/L). Sham (CTRL diet: n=5, HFD: n=4), moderate lesion (CTRL diet: n=3, HFD: n=3) and severe lesion (CTRL diet: n=6, HFD: n=4). Two-way ANOVA: p* <0.05, p**<0.01, p***<0.001, p****<0.0001. GTT = glucose tolerance test, AUC = area under the curve.
