# Supplementary material for: High-fat diet-induced diabetes leads to vascular alterations, pericyte reduction, and perivascular depletion of microglia in a 6-OHDA toxin model of Parkinson disease
Source: J Neuroinflammation. 2021 Aug 10;18:175. doi: 10.1186/s12974-021-02218-8 (PMC8353816; doi:10.1186/s12974-021-02218-8)
Supplement: Supplementary file 2 — Additional file 2: Figure 2. Diet effect on the lipid content in the liver and plasma leptin level. (A) Bright field images showing ORO+ lipid staining in the liver of sham, moderate and severe lesion group fed with either CTRL diet or HFD. (B) corresponding quantification of ORO density in the liver. Data are expressed in % of total image area. Sham (CTRL diet: n=4, HFD: n=3), moderate lesion (CTRL diet: n=1, HFD: n=1) and severe lesion (CTRL diet: n=5, HFD: n=3). (C) Plasma leptin level of sham, moderate and severe lesion group fed with either CTRL diet or HFD. Data are expressed as pg/ml. Sham (CTRL diet: n=3, HFD: n=4), moderate lesion (CTRL diet: n=1, HFD: n=3) and severe lesion (CTRL diet: n=3, HFD: n=3). Two-way ANOVA: p* <0.05, p**<0.01, p***<0.001, p****<0.0001. [file 12974_2021_2218_MOESM2_ESM.pdf]

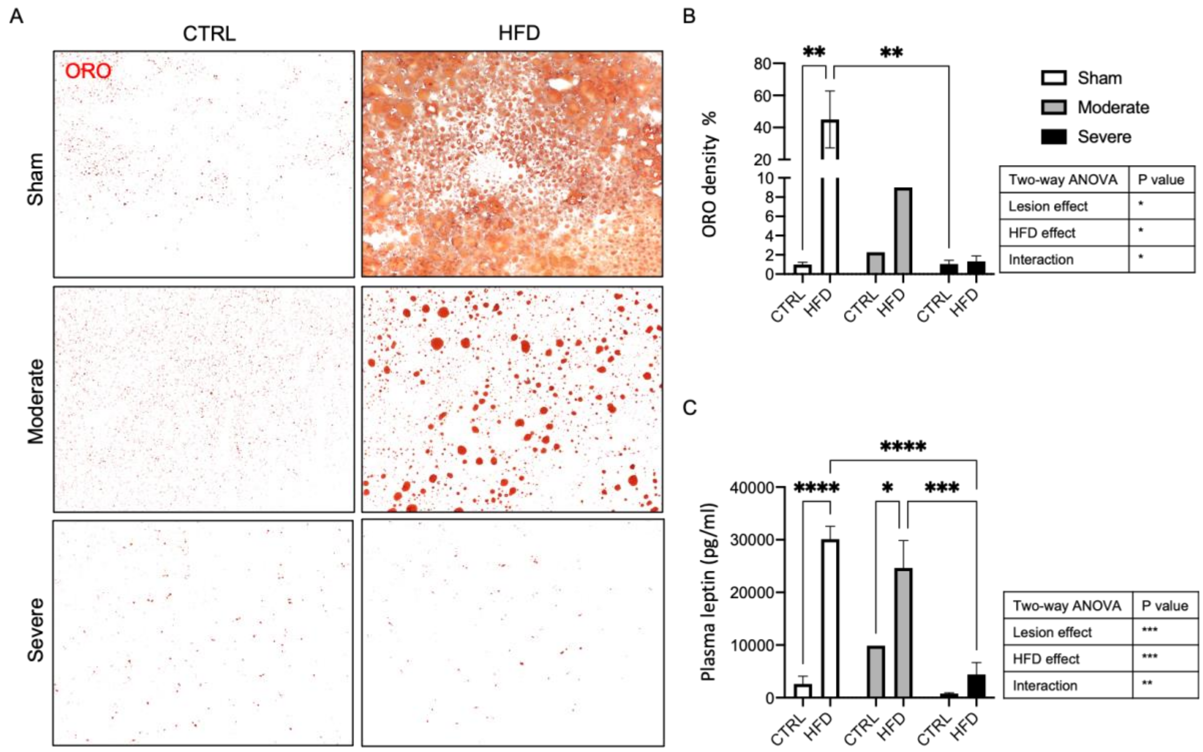

Additional figure 2: Diet effect on the lipid content in the liver and plasma leptin level. (A) Bright field images showing ORO$^+$ lipid staining in the liver of sham, moderate and severe lesion group fed with either CTRL diet or HFD. (B) corresponding quantification of ORO density in the liver. Data are expressed in % of total image area. Sham (CTRL diet: n=4, HFD: n=3), moderate lesion (CTRL diet: n=1, HFD: n=1) and severe lesion (CTRL diet: n=5, HFD: n=3).

(C) Plasma leptin level of sham, moderate and severe lesion group fed with either CTRL diet or HFD. Data are expressed as pg/ml. Sham (CTRL diet: n=3, HFD: n=4), moderate lesion (CTRL diet: n=1, HFD: n=3) and severe lesion (CTRL diet: n=3, HFD: n=3). Two-way ANOVA: p* <0.05, p**<0.01, p***<0.001, p****<0.0001.
