# Supplementary material for: High-fat diet-induced diabetes leads to vascular alterations, pericyte reduction, and perivascular depletion of microglia in a 6-OHDA toxin model of Parkinson disease
Source: J Neuroinflammation. 2021 Aug 10;18:175. doi: 10.1186/s12974-021-02218-8 (PMC8353816; doi:10.1186/s12974-021-02218-8)
Supplement: Supplementary file 3 — Additional file 3: Figure 3. Linear regression analysis between the CD11b+/CD31+ interaction density and the CD13+ pericyte density of sham, moderate and severe lesion group fed with either CTRL diet or HFD. Sham mice (CTRL diet: n=5, HFD: n=4), moderate lesion (CTRL diet: n=3, HFD: n=3) and severe lesion (CTRL diet: n=6, HFD: n=4). p**<0.01 [file 12974_2021_2218_MOESM3_ESM.pdf]

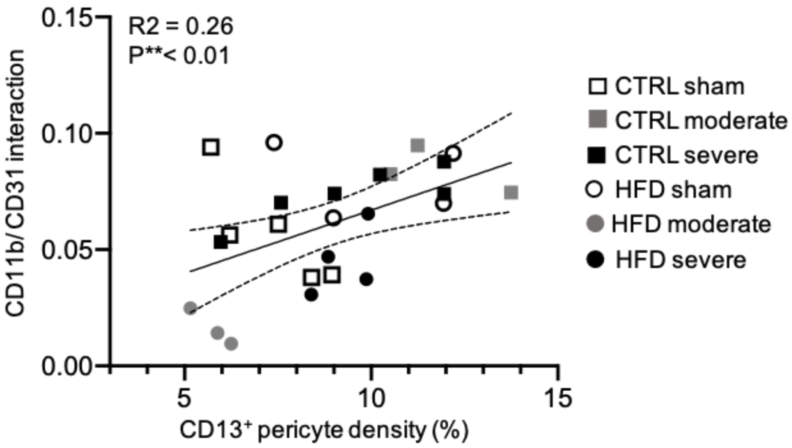

Additional figure 3: Linear regression analysis between the $CD11b^+/CD31^+$ interaction density and the $CD13^+$ pericyte density of sham, moderate and severe lesion group fed with either CTRL diet or HFD. Sham mice (CTRL diet: n=5, HFD: n=4), moderate lesion (CTRL diet: n=3, HFD: n=3) and severe lesion (CTRL diet: n=6, HFD: n=4). p**<0.01.
